# Supplementary material for: Bimodular Antiparallel G-Quadruplex Nanoconstruct with Antiproliferative Activity
Source: Molecules. 2019 Oct 8;24(19):3625. doi: 10.3390/molecules24193625 (PMC6803823; doi:10.3390/molecules24193625)
Supplement: Supplementary file 1 [file molecules-24-03625-s001.pdf]

molecules

Supplementary Information

Table S1. Circular dichroism melting temperatures and percentage of folded fraction at 37°C for DNA oligonucleotides under study

No.	Name	Circular Dichroism Tm, °C	Percentage of folded ODN at 37C, %
1	HD1	39.2 [1], 49.1 [2]	>50%
2	biHD1	32 [1]	<50%
3	biHD1-C3	37	50
4	biHD1+Ba	-	-
5	biHD1-T4A,T20A	39	61
6	biHD1-5′-Δ2G	35	47

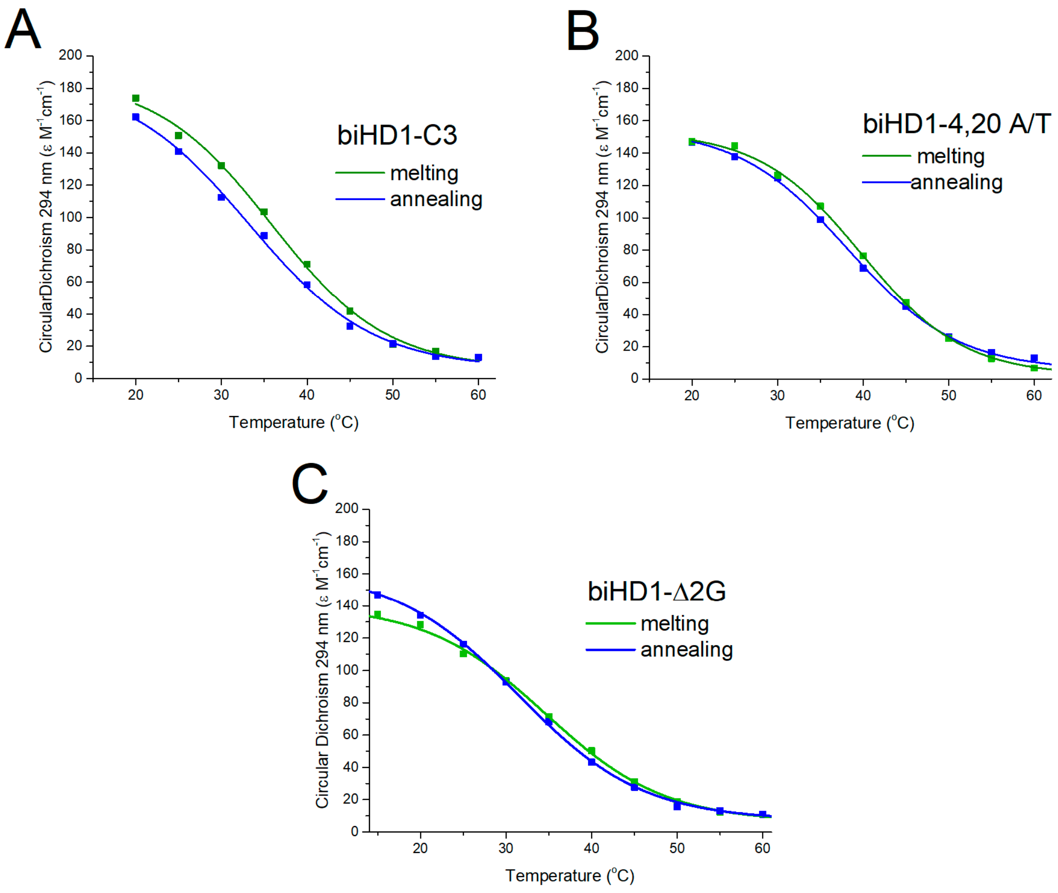

Figure S1. CD-melting (green) and annealing (blue) curves are plotted as functions of biHD1-T4A,T20A (**A**), biHD1-C3 (**B**) and biHD1-Δ2G (**C**) amplitude at 294 nm of temperature. CD data were recorded in a cuvette with a path length of 1 cm in the temperature range of 20–70°C with a scan rate of 30°C/h at 2.5 μM ODN strand concentration in working buffer solution (10 mM NaH_2PO_4/Na_2HPO_4, 10 mM KCl, 140 mM NaCl, pH 7.5).

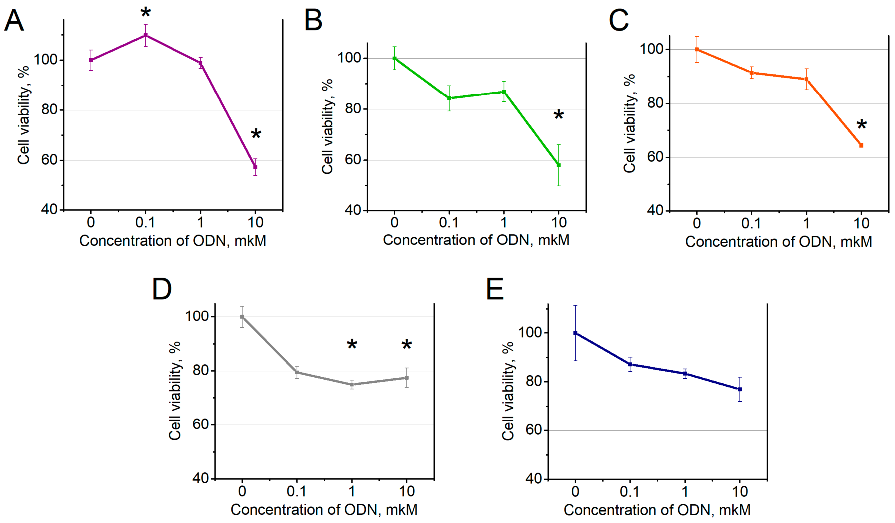

Figure S2. MTT test results of biHD1 treatment for different cell lines: **A** - HeLa, **B** - HCT116, **C** - MCF7, **D** - mS, **E** - PC3. Cells were incubated for 72 hr without oligonucleotide, with 0.10 μM, 1.0 μM, and 10 μM biHD1 oligonucleotide. Data are presented as mean ± S.D. Asterisk - $p < 0.05$ compared with control (w/o oligo).

Table S2. Investigation of biHD1 IC50 for different cell lines

Cell Line	biHD1 IC50, μM
RL-67	1
U87	7
HeLa	>10
HCT116	>10
MCF7	>10
mS	>10
PC3	>10
hEF	>10

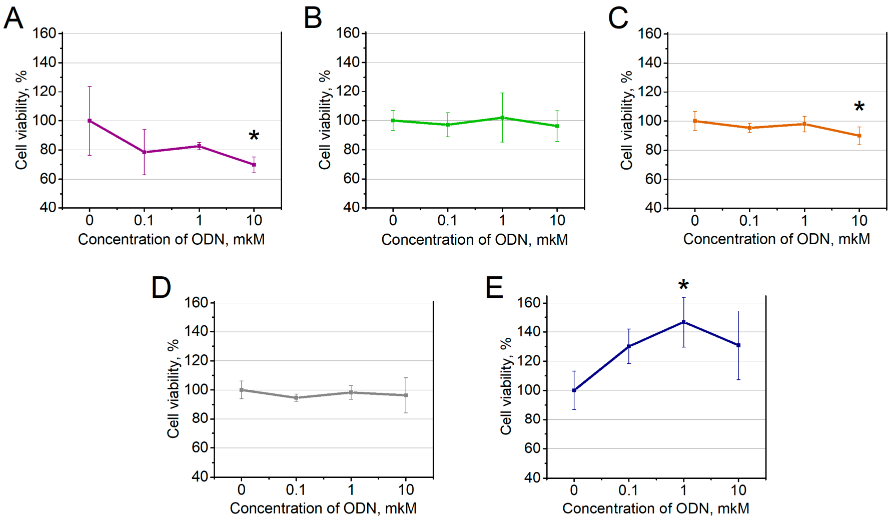

Figure S3. MTT test results of biHD1-C3 treatment for different cell lines: **A** - HeLa, **B** - HCT116, **C** - MCF7, **D** - mS, **E** - PC3. Cells were incubated for 72 hr without oligonucleotide, with 0.10 μM, 1.0 μM, and 10 μM biHD1-C3. Data are presented as mean ± S.D. Asterisk - $p < 0.05$ compared with control (w/o oligo).

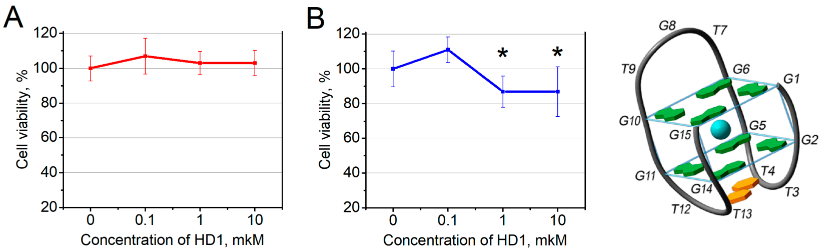

Figure S4. MTT test results for U87 (A) and embryo fibroblast (B) cell lines after 72 hr incubation of cells with different concentrations of single-modular HD1: control experiment without oligonucleotide, 0.10 μM HD1, 10 μM oligonucleotide, and 10 μM oligonucleotide. Data are presented as mean ± S.D. Asterisk - $p < 0.05$ compared with control (w/o oligo).

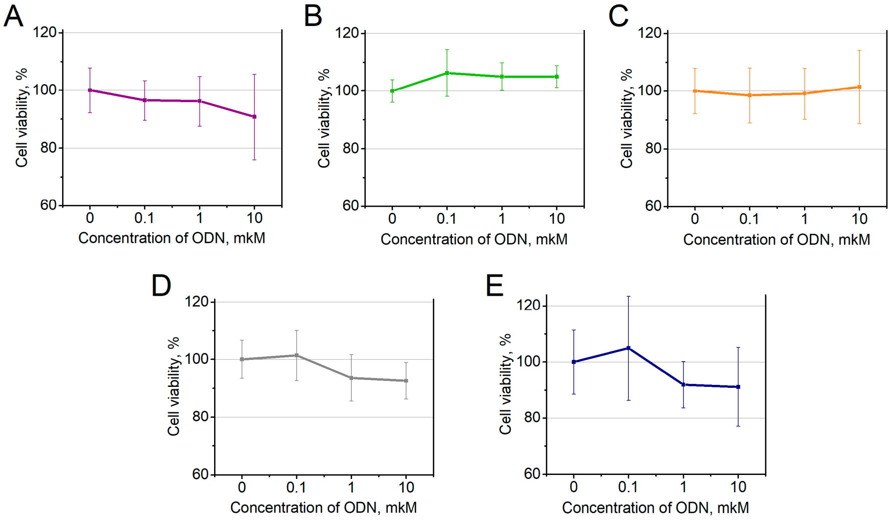

Figure S5. MTT test results of biHD1-A4TA20T treatment for different cell lines: **A** - HeLa, **B** - HCT116, **C** - MCF7, **D** - mS, **E** - PC3. Cells were incubated for 72 hr without oligonucleotide, with 0.10 µM, 1.0 µM, and 10 µM biHD1-A4T, A20T. Data are presented as mean ± S.D. Asterisk - $p < 0.05$ compared with control (w/o oligo).

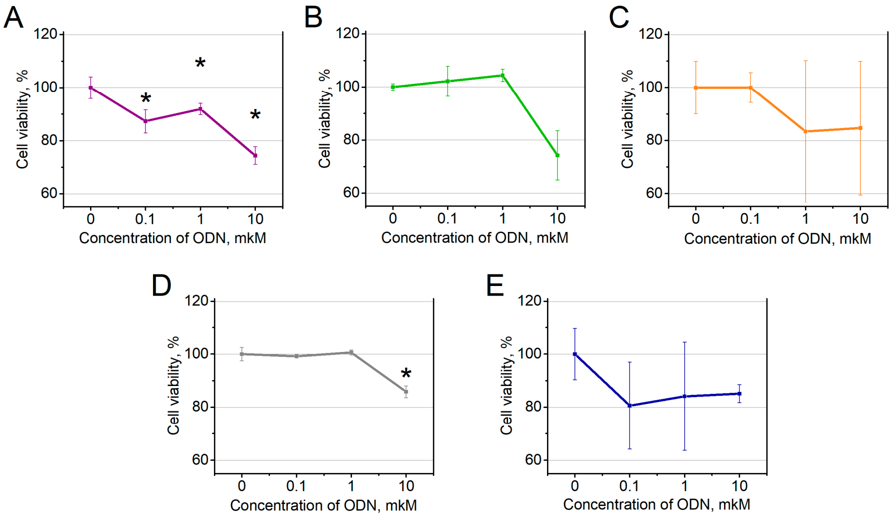

Figure S6. MTT test results of biHD1-5′-Δ2G treatment for different cell lines: **A** - HeLa, **B** - HCT116, **C** - MCF7, **D** - mS, **E** - PC3. Cells were incubated for 72 hr without oligonucleotide, with 0.10 µM, 1.0 µM, and 10 µM biHD1-5′-Δ2G. Data are presented as mean ± S.D. Asterisk - $p < 0.05$ compared with control (w/o oligo).

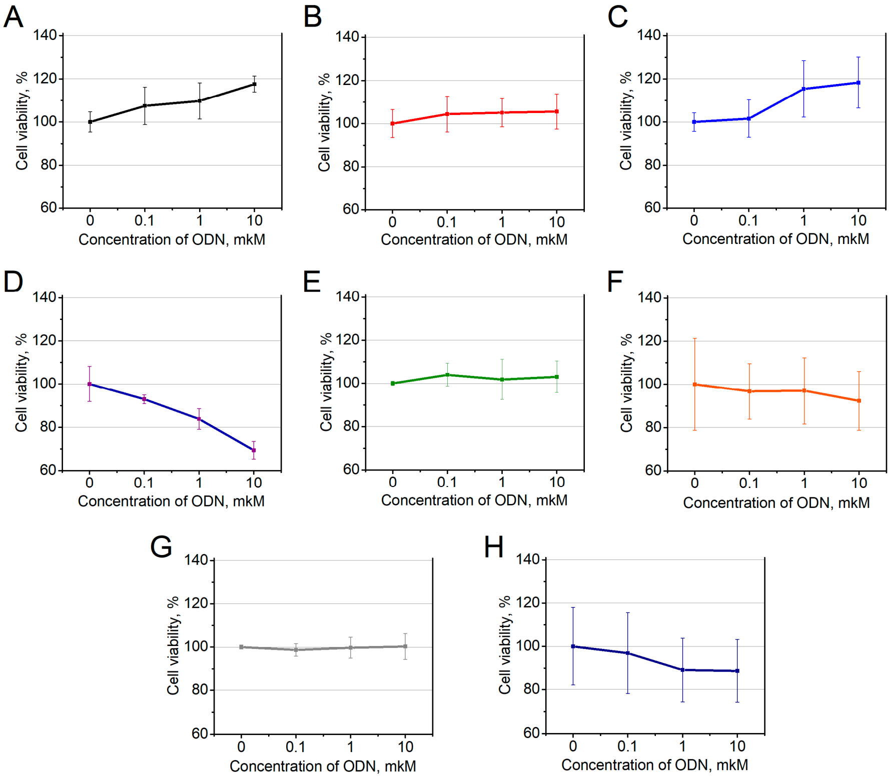

Figure S7. MTT test results of biHD1+Ba treatment for different cell lines: **A** - RL-67, **B** - U87, **C** - embrionic fibroblasts, **D** - HeLa, **E** - HCT116, **F** - MCF7, **G** - mS, **H** - PC3. Cells were incubated for 72 hr without oligonucleotide, with 0.10 μM, 1.0 μM, and 10 μM biHD1+Ba. Data are presented as mean ± S.D.

molecules

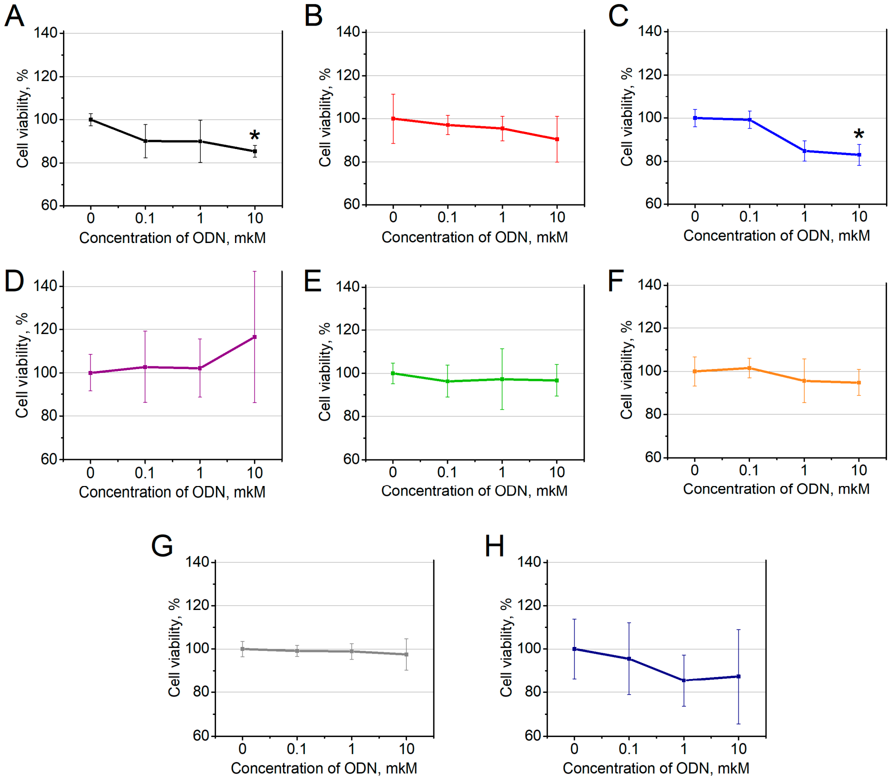

Figure S8. MTT test results of $BaCl_2$ treatment for different cell lines: **A** - RL-67, **B** - U87, **C** - embrionic fibroblasts, **D** - HeLa, **E** - HCT116, **F** - MCF7, **G** - mS, **H** - PC3. Cells were incubated for 72 hr without salt, with 0.20 µM, 2.0 µM, and 20 µM $BaCl_2$. Data are presented as mean ± S.D. Asterisk - $p < 0.05$ compared with control (w/o oligo).

References

1. Amato, T., Virgilio, A., Pirone, L., Vellecco, V., Bucci, M., Pedone, E., Esposito, V., Galeone, A. Investigating the properties of TBA variants with twin thrombin binding domains. Sci Rep. **2019**. *9*(1):9184. doi: 10.1038/s41598-019-45526-z.

2. Zavyalova, E.G.; Legatova, V.A.; Alieva, R.S.; Zalevsky, A.O.; Tashlitsky, V.N.; Arutyunyan, A.M.; Kopylov, A.M. Putative mechanisms underlying high inhibitory activities of bimodular DNA aptamers to Thrombin. Biomolecules. **2019** *9*(2), E41. doi: 10.3390/biom9020041
